# The effect of mentorship program in enhancing the academic performance of first MBBS students

**DOI:** 10.30476/jamp.2019.82591.1061

**Published:** 2020-10

**Authors:** NAGA GUHAN, PRAKASH KRISHNAN, PRIYA DHARSHINI, PHILIP ABRAHAM, SUNI THOMAS

**Affiliations:** 1 Department of Biochemistry, All India Institute of Medical Sciences, Mangalagiri, India; 2 Department of Pharmacology, Al-Azhar medical college, Kerala, India; 3 Department of Anatomy, Alluri Sitarama Raju Academy of Medical Sciences, Andhra Pradesh, India; 4 Department of Biochemistry, Al Azhar medical college, Kerala, India

**Keywords:** Mentorship, Medical education, Academic performance

## Abstract

**Introduction:**

Mentoring is a natural process, which blossoms from the desire of experienced veterans to give and the thirst of inexperienced novices to grow. Formal faculty mentoring could help the 1^st^ year students to thrive on the complicated situations in a newer environment and excel in their career. This study was designed to evaluate the effectiveness of mentorship program in improving the academic performance of 1^st^ MBBS students.

**Methods:**

148 first MBBS students of Al Azhar medical college, who were admitted for 2017-18 academic year, were included for this interventional study. Mentorship program was started in our college since January 2018 after first internal exam, wherein the students were equally divided among 6 mentors. At the end of 6 months, to assess the effectiveness of mentorship program, a post-exam was conducted and the marks were compared with the pre-program performance. Paired t test was used to compare the marks before and after the program. Furthermore, the perception of mentees on mentorship program was also assessed by a valid questionnaire (score of 1-4).

**Results:**

The mean score of students in the exam conducted after mentorship program was significantly higher (p<0.001) than that conducted before commencing mentorship program. This increment in performance is appreciated more in girls rather than the boys. Furthermore, the mentorship program significantly (p<0.001) helped to boost the academic performance in below average students who had scored < 50% marks in pre-program assessment. The effectiveness of the mentorship program was further supported by the students’ feedback. 40.5% of the students agreed and 56.8% of them strongly agreed that mentorship program was effective and beneficial to them.

**Conclusion:**

The mentorship program obviously improves the academic performance of students, especially below average performers who need extra care and guidance.

## Introduction

Mentorship program is a process whereby an experienced, highly regarded, empathetic person (the mentor) guides another (usually younger) individual (the mentee) in the development and re-examination of their own ideas, learning, and personal and professional development ( 1 ). It blossoms from the desire of an experienced veteran to give and the thirst of an inexperienced novice to grow. It helps to provide effective academic & psychological support and guidance to students that contribute to successful undergraduate training ( 2 ). 

First year MBBS students are exposed to lot of obstacles, which may prevent them from excelling in academics. They have to face a new environment and new challenges in medical course, which force them to seek for an extra care and guidance, so that they can cope up with the difficulties faced in a new environment. Formal faculty mentoring could help the students to thrive on the complicated situations they face in the initial stages and excel in their career.

Though mentorship programs were developed in the USA in the 1970s, formal mentoring programs for medical students and doctors, however, were not developed until the late 1990s ( 3 ). There is growing evidence for the positive effects of mentoring programs in undergraduate medical education in professional and personal development. In a prospective study on career development in young physicians, graduates stated that mentoring in medical school would have helped them to make their decision on specialty training earlier and to adopt a more goal-oriented strategy in planning their careers. 

Apart from students, the teachers, institute & society are also benefitted from this program ( 4 ). It provides a sense of internal satisfaction and thus helps in personal development of the faculty. In addition, it also improves clinical care, research as well as teaching. Therefore this study is designed to assess the effects of mentorship program among first MBBS students. The aims of this study were to study the effect of mentorship program in enhancing the academic performance of first MBBS students and to assess the perception of mentees on effectiveness of mentorship program using a questionnaire. 

## Methods

This single group experimental study was conducted in the Department of Medical Education, Al - Azhar Medical College, Kerala. Excluding two students who were absent for first or last internal exam, we enrolled the remaining 148 first year students admitted in the academic year 2017-2018 for this study. The mentorship program started in the college in January, 2018 after the first mid-semester exam. The students were divided among 6 faculty members who were sensitised to the guidelines and procedure of mentorship program before commencement of the program. The students were divided according to their roll numbers and they were also sensitised regarding their role as mentees. During the mentorship program, each student was spending a minimum of half an hour per week with the assigned mentor. At the end of 6 months, after obtaining approval from IRB, the exam was conducted and the marks were compared with pre-program performance. Furthermore, the perception of mentees on mentorship program was also assessed by a questionnaire in a scale of 1-5. After obtaining data, the mean marks of the students before and after the program were compared using paired t test. Chi-square test was used to find association between two variables. A p value≤0.05 was considered as statistically significant. SPSS 20.0 software was used for all statistical analyses.

## Results

The students scored significantly (p< 0.001) higher marks in the exam conducted after the mentorship program compared to those before commencing the program. This increment in performance is appreciated more in the girls rather than in the boys. Furthermore, the mentorship program significantly (p<0.001) helped to boost the academic performance in below average students who had scored < 50% marks in pre-programme assessment.(Table 1) The effectiveness of mentorship program was further supported by the students’ feedback. 40.5% of the students agreed and 56.8% of them strongly agreed that mentorship program was effective and beneficial to them. (Table 2)

**Table 1:** Comparison of marks before and after mentorship program

	Number of students	Marks before Mentorship (Mean±SD)	Marks after Mentorship (Mean±SD)	p value
Total students	148	22.8±8.1	25.3±7.7	<0.001
Girls	108	24.5±7.6	27.4±6.8	<0.001
Boys	40	18.2±7.4	19.4±7.3	0.273
Students who scored >50% in first internal exam	73	29.4±4.1	30.2±5.7	0.221
Students who scored <50% in first internal exam	75	16.3±5.1	20.5±6.4	<0.001

**Table 2:** Students’ feedback for mentorship program

	Questions	Strongly Disagree (1)	Disagree (2)	Agree (3)	Strongly Agree (4)
1	My mentor was accessible and available.	0%	4.1%	37.8%	58.1%
2	My mentor communicated regularly with me.	0%	19.6%	52.7%	27.7%
3	My mentor assisted me with my career queries.	1.3%	11.5%	50.7%	36.5%
4	Mentorship program assisted me in improving my course work performance.	0%	6.8%	37.8%	55.4%
5	Mentorship program assisted me with my understanding of the academic routes to achieve my current career goals.	0%	7.4%	41.2%	51.4%
6	My mentor demonstrated a reasonable interest/concern towards me.	2.1%	7.4%	55.4%	35.1%
7	My mentor’s behavior and attitude is generally an example of professionalism.	0%	8.8%	36.5%	54.7%
8	I learned at least one important lesson about my career or professionalism from my mentor.	0%	7.4%	38.5%	54.1%
9	I recommend mentorship program for future professional or personal development activities.	1.3%	1.3%	33.1%	64.2%
10	Overall, mentorship program was effective and beneficial to me.	0.6%	2.1%	40.5%	56.8%

## Discussion

Mentoring is defined as an insightful process in which the mentor's wisdom is acquired and modified as needed by the mentee, as well as a process that is supportive and often protective ( 5 ). Mentorship program is acknowledged as an important career advancement tool and a key to successful and satisfying careers ( 3 ). Unlike teachers or tutors, mentors actively help the mentees to augment their potential and to conquer their personal and professional goals ( 6 ). The five different phases in mentoring includes information on career options, developing career plans, focusing on career goals, realization of career steps, and evaluation of career advancement. Frei, et al. have summarised in their work that the key objectives of mentorship program should be the development of professionalism and personal growth, the promotion of interest in academics, and the provision of career counselling ( 7 ). 

With similar objectives, mentorship program was introduced in our institute for the first MBBS students. The faculty members were sensitised to the guidelines and procedures of mentorship program and they were elaborated on their role and importance of delivering effective feedback before commencement of the program. Likewise, the mentees were also made aware of their role and what is expected of them. Protected time was dedicated to mentoring activities to encourage interaction and engagement between the mentors and the mentees. Among 150 students, two students who were absent for internal exams, were excluded from this study. For the remaining 148 students, mentorship program proved to maximise their academic performance and its effectiveness was more evident among the below average students. 

In concordance with our present study, various research works provided evidence to showcase the effectiveness of mentorship program. According to Levinson W, et al. mentorship program enhances the productivity, career advancement and career satisfaction ( 8 ). Applegate and Williams, in their research, proved that mentoring offers personal and professional benefits to mentees ( 9 ). A study by Keating LM, et al. supported the positive influence of mentoring on at-risk youth ( 10 ). Erickson, et al., in their study, found a great impact of mentors on overall educational achievement of mentees ( 11 ). According to Hawkins A., et al. the key benefits of mentorship program were academic support, improved confidence, increased enjoyment and sense of belonging ( 12 ). 

Apart from these benefits, V Devi, et al. in their work, found that mentorship program had the ability to inculcate research skills in students and encourage the development of positive attitude and knowledge about scientific research ( 13 ). In our study, the program was less effective in certain subsets of students like boys and in students with good grades. The probable reasons for this could be lack of initiative, time and commitment from both the ends, as revealed by Bhatia A, et al. ( 14 ). Most of these studies used mainly surveys or questionnaires to evaluate the program, whereas in the current research, apart from assessing the students’ satisfaction and experiences, we also evaluated a tangible outcome - exam marks. The extension of this mentorship program and evaluation on frequent basis with continuous improvements can offer long term benefits ( 15 ). 

### Limitations

More than academic performance, it is emotional quotient, which is directly influenced by mentorship program. The influence of mentorship program on emotional stability of students might be taken into considerations.

## Conclusion

The mentorship program obviously improved the academic performance of students especially in below average performers, who needed extra care and support. The benefits of mentorship program are not only limited to academic performance but also to emotional and personal aspects including career development, improved relationships with faculty, greater interest in research, aspirations toward academic careers, improved self-esteem and reduced stress. In future, the study can be extrapolated to assess the other benefits, too. 
